# Challenges for Plant Growth Promoting Microorganism Transfer from Science to Industry: A Case Study from Chile

**DOI:** 10.3390/microorganisms11041061

**Published:** 2023-04-19

**Authors:** Eduardo Muñoz-Carvajal, Juan Pablo Araya-Angel, Nicolás Garrido-Sáez, Máximo González, Alexandra Stoll

**Affiliations:** 1 Laboratorio de Microbiología Aplicada, Centro de Estudios Avanzados en Zonas Áridas, La Serena 1720256, Chile; 2 Departamento de Biología, Facultad de Ciencias, Universidad de La Serena, La Serena 1720256, Chile; 3 Instituto de Investigación Multidisciplinario en Ciencia y Tecnología, Universidad de La Serena, La Serena 1720256, Chile

**Keywords:** inter- and transdisciplinary research, collaborative innovation, geographic inequality, natural laboratory

## Abstract

Research on the plant growth promoting microorganisms (PGPM) is increasing strongly due to the biotechnological potential for the agricultural, forestry, and food industry. The benefits of using PGPM in crop production are well proven; however, their incorporation in agricultural management is still limited. Therefore, we wanted to explore the gaps and challenges for the transfer of biotechnological innovations based on PGPM to the agricultural sector. Our systematic review of the state of the art of PGPM research and knowledge transfer takes Chile as an example. Several transfer limiting aspects are identified and discussed. Our two main conclusions are: neither academia nor industry can meet unfounded expectations during technology transfer, but mutually clarifying their needs, capabilities, and limitations is the starting point for successful collaborations; the generation of a collaborative innovation environment, where academia as well as public and private stakeholders (including the local community) take part, is crucial to enhance the acceptance and integration of PGPM on the way to sustainable agriculture.

## 1. Introduction

Around the world, research on the beneficial plant-microorganisms interaction is becoming more attractive due to the biotechnological potential for the agricultural, forestry, and food industry [1,2]. These “plant growth promoting microorganisms” (PGPM) interact in symbiosis with plants, playing important functional roles in germination, development, and adaptation to environmental characteristics [3]. During the last decades, dozens of microbial species and hundreds PGPM strains have been studied by research groups worldwide. Scientific publications disclose their characteristics and performance in interactions with plants under laboratory, greenhouse, and field conditions [4,5,6]. Often these strains are very well characterized and scientific knowledge encompasses background on their identity, growth conditions, colonization ability, incompatibilities, as well as primary and secondary metabolism. More advanced studies may complement genetic data or identification of specific secondary metabolites. This information is highly relevant for the development of microbial inoculants. 

Despite the diversity of plant associated microorganisms, the most commonly studied PGPM are *Rhizobium*, *Pseudomonas*, *Azospirillum*, *Azotobacter, Bacillus*, *Glomus*, and *Trichodermas* [7,8]. Some strains of *Pseudomonas*, *Rhizobium*, *Bacillus*, *Glomus*, and *Trichoderma* are also commercially available as single or combined strains.

**Figure 1:**
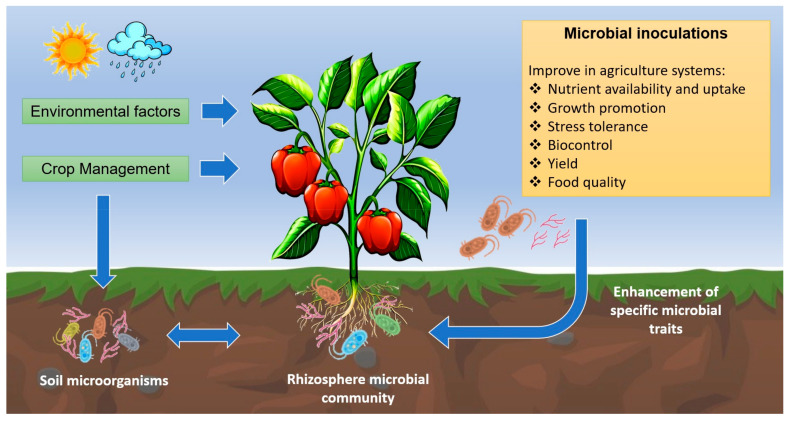
Graphical summary of PGPM services for agricultural production.

Applied studies highlight their benefits for agricultural production generated by the inoculation of beneficial microorganisms in crop plants (Figure 1) [9]. Such benefits are increased productivity and crop yield (e.g., a major fruit number, major plant size, plant survival, and increases in germination rate) [9,10]. Additionally, PGPM increases the plant’s tolerance against biotic and abiotic stress, an important characteristic for facing the challenges of global change in agriculture and forestry [10]. The use of beneficial microorganisms in agriculture is considered as environmentally friendly, as the application of chemical fertilizers and pesticides can be significantly reduced [9,11]. 

An effective use of PGPM in agriculture could contribute to combating undernourishment and food insecurity as well as to optimize agricultural inputs in energy, fertilization, pest control, and water [11,12]. The benefits of using PGPM in crop production are well proven; however, their incorporation in agricultural management is still limited. Therefore, we wanted to explore the gaps and challenges for the transfer of biotechnological innovations based on PGPM to the agricultural sector. Our systematic review on the state of the art of PGPM research and knowledge transfer takes Chile as an example. Chile is considered as a natural laboratory, stretching out from subtropical to subantarctic ecosystems. This particularity transforms it into a model for extrapolating to global tendencies [13].

## 2. Materials and Methods

### 2.1. Study Case 

Chile has a surface area of 756,950 km^2^, with many climates, including the driest desert on Earth (Atacama Desert), Mediterranean areas, rainforests, and southern areas such as Antarctica. In addition, Chile presents varied geography characterized by the mountain ranges of the Andes and the coastal mountain range, the Atacama Desert, the altiplano, central valleys, and extensive pampas in Patagonia [14]. 

### 2.2. Search Strategy for Scientific Publications 

A bibliographic search was carried out in PubMed, Web of Science, and Scopus databases. Keywords related to beneficial plant-microorganism interaction in Chile were used to identify articles published up to 31 May 2021. On the other hand, we searched for articles from leading research groups in this field published in the same time frame. Please refer to the Supplementary Material for more details (Figure S1). Duplicates and grey literature, book chapters etc. were eliminated.

### 2.3. Selection of Scientific Publications

Different selection criteria were considered to select articles that fit the objectives (Figure S1). Only articles describing, isolating, or applying beneficial microorganisms associated with plants in Chile were selected, excluding articles on plant pathogens and reviews.

### 2.4. Meta-Analysis

For the spatial analysis, the geographic information (sampling or study area) was extracted from all selected articles and classified into six geographic zones based on available information on the agro-climatic atlas of Chile [15]. The geographic zones are denominated as: zone I: hyper-arid zone (18 to 25° S), zone II: arid to semi-arid zone (25° to 32° S), zone III: Mediterranean zone (32° to 36° S), zone IV: temperate (warm) zone (36° to 39° S), zone V: temperate (cold) zone (39° to 44° S), zone VI: Patagonian to subantarctic zone (44° to 55° S) (Table S1). The articles were analyzed according to the type of technique (culture-independent, culture-dependent, and both), origin and type of microorganism, and plant host (commercial, native, or both). 

Additional information was extracted to further characterize the selected research studies (Supplementary Material 2):

(1) Type of PGP mechanism: here, we distinguished 7 PGPM mechanisms (phosphate solubilization, N fixation, siderophores production, phytohormones production, ACC-deaminase activity, exopolysaccharides production, biocontrol capacity), as well as the category “others” for all other reported mechanisms.
(2) Type of environmental stress: here, we distinguished pH, nutrient deficit, salinity, drought, pollution, temperature (heat or cold), UV radiation, altitude, herbivory, fire, and others.
(3) Inoculated plant type (vegetable, grass, fruit tree, etc.).
(4) Technology Readiness Level (TRL) [16].
(5) Strain identity.

### 2.5. Database of Microorganisms Available for the Chilean Agricultural Sector

The record of officially registered commercial strains and products in Chile and the registry of the commercializing companies was taken from the report of the Agricultural and Livestock Service of the Chilean Ministry of Agriculture (January 2021).

## 3. Plant-Microorganism Interaction in Chile: A Case Study 

### 3.1. General Overview of Plant-Microorganism Interaction in Chile

**Figure 2:**
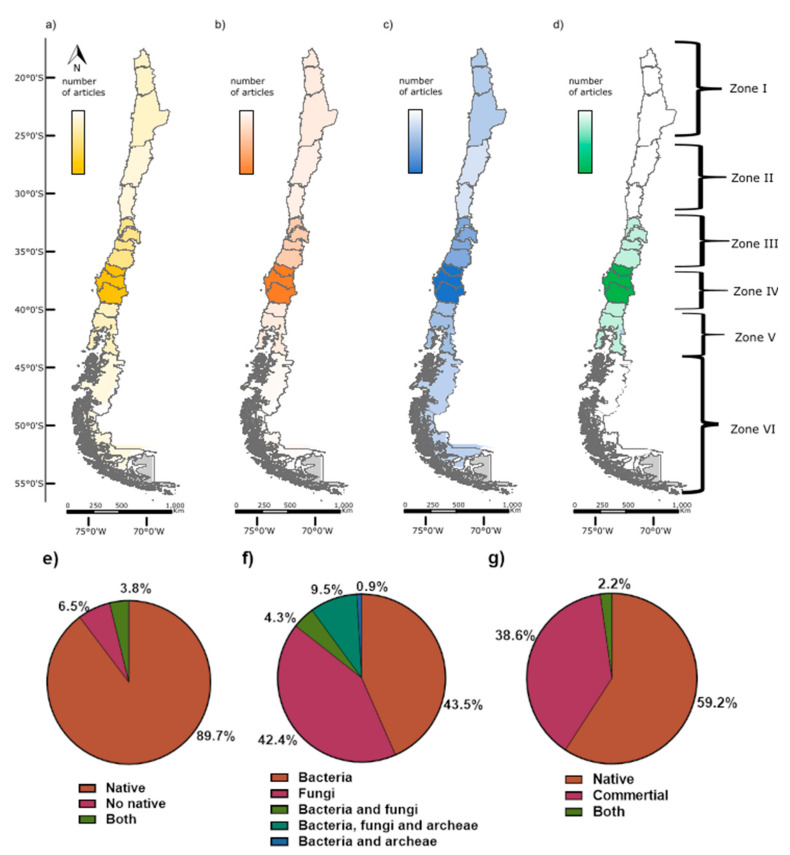
Geographical distribution of the PGPM studies in Chile and general study focus. (**a**) Spatial distribution of all articles (183) in this review; (**b**) articles based on culture-dependent techniques (105); (**c**) articles based on culture-independent techniques (71); (**d**) articles considering both type techniques (7); (**e**) Biogeographic origin of the microorganisms in all articles; (**f**) Taxonomic identity of the microorganism (domain level) in all articles; (**g**) Biogeographic origin of the plant host in all articles. The total number of articles is 183. (For the description of zone I to zone VI, please refer to methodological Section 2.4).

Our final database contained 183 scientific publications on PGPM studies realized in Chile between 1989 and 2021. However, it was only in the year 2000 that the number of publications increased significantly (Figure S2). Most studies applied culture-dependent techniques to characterize PGPM-plant interaction (104 articles, Figure 2, Table S2). Nonetheless, studies based on culture-independent techniques have had greater importance since 2010 (Figure S1) due to the massification of next-generation sequencing techniques, bioinformatics analysis of large amounts of data, and the availability of large databases [17,18]. This technical progress enabled an increased resolution of the plant-associated microbial diversity. Interestingly, very few studies employed a combination of culture-dependent and -independent techniques (Figure S2).

Globally, articles are not evenly distributed across geographic zones. Together, the warm-temperate and Mediterranean zone (zone III and IV) comprise approximately 2/3 of the analyzed articles (45.65% (83) and 20.65% (38), respectively) (Figure 2a). These two zones only represent 15% of the national territory but are the richest biodiversity area and contain a high diversity of ecosystems [19]. Furthermore, environmental conditions such as higher precipitation and fertile soils [20,21] favor human settlement (48.9% of the Chilean population) and general development (e.g., agriculture, research) in the warm-temperate and Mediterranean zone [22].

In contrast, geographic zones located in the extreme north (Zone I and II) and south of Chile (Zone V and VI) present a substantially lower number of articles on PGPM (9.78% (18), 6.52% (12), 11.41% (21), and 5.98% (11), respectively). These environments are known for complex and extreme conditions in terms of salinity, aridity, UV radiation, temperature, pH, and heavy metals [23], limiting agricultural activities [24]. Nevertheless, environments may harbor beneficial microorganisms, which can contribute to a quick adaptation of agriculture to climate change conditions such as drought, increased UV, salinity, cold, and heat [25,26], without the replacement to change crop varieties. However, this potential is poorly explored as only 27% of the culture-dependent studies were performed in these geographic zones (zone I and II, zone V and VI) (Figure 2b).

For articles based on culture-independent techniques, the distribution tends to be slightly less biased towards the warm-temperate and Mediterranean zone (Figure 2c), where still 54% of the studies were carried out.

Only seven articles used culture-dependent and independent techniques, of which 5 were carried out in the zone with the highest article number, Zone IV (Figure 2d).

In 89.7% of the revised articles, the studied microorganisms were native to Chile, while only 6.5% used strains introduced (Figure 2e). The effectiveness of introduced microorganisms has been discussed [7,11]. Some studies address this issue, such as Salvatierra-Martinez et al. (2015) [27], where tolerance to salinity and antagonistic capacity were evaluated in 10 native *Trichoderma* spp. Strains and a commercial bioformulation of exogenous. The native strains demonstrated more tolerance to salinity and better performance in plant growth promotion.

Most studies focus on bacteria or fungi, mainly mycorrhizae (43.5% and 42.4% of the articles, respectively), whereas 9.2% included both microorganisms (Figure 2f).

Finally, 59.2% of the revised articles work with a native plant host, whereas 38.6% evaluate the microbial effect on plants for commercial use, such as agricultural and forestry crops (Figure 2g).

PGPM present a wide range of mechanisms to interact with the plant host [28], including nutrient availability for the host, biocontrol, phytohormone production, etc. In our data set, the most studied mechanisms are biocontrol capacity and phosphate solubilization (20 and 9 articles, respectively). Nonetheless, most studies display a very narrow characterization of the investigated microorganisms, with more than 80% of the articles focusing on one mechanism. Only a small number of studies evaluate experimentally two or more mechanisms (16%). This high specificity regarding the studied PGPM mechanisms does not allow for assessing the capabilities of the isolated strains in a more holistic approach, even though this kind of information would be very helpful for selecting promising microbial strains. Thus, our understanding of the distribution, acquisition, or loss of functional traits in PGPM as an adaption to the plant host niche is still very limited. 

### 3.2. Reduction of Environmental Stress through Inoculation of Microorganisms

Several consequences of global change are challenging for the agricultural sector, particularly when these increase the abiotic stresses for the crop plants, such as floods, salinity, soil degradation, high temperature, and drought [29]. Different microbial mechanisms have been described to improve plant growth under stress conditions, such as the production of phytohormones and exopolysaccharides, as well as the activity of the ACC deaminase enzyme [7,28,30]. In this sense, we searched our database for articles that evaluate the interaction of PGPM and their plant host under environmental conditions.

**Figure 3:**
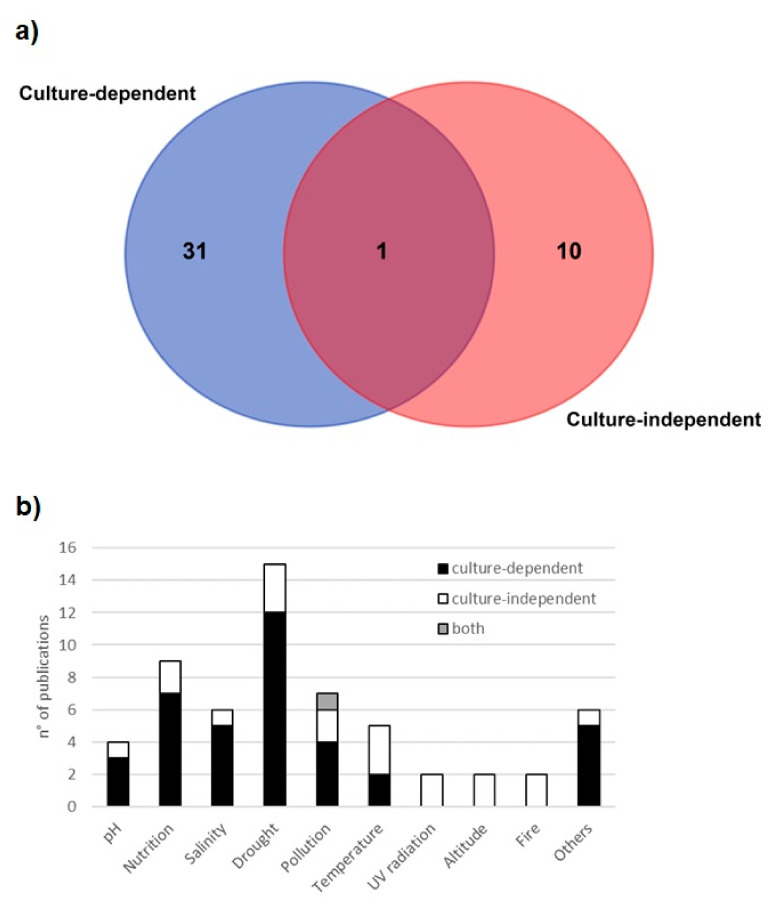
Publications with a research focus on PGPM + environmental stress + stress mitigation. (**a**) Venn diagram with the number of articles per study approach, (**b**) the number of articles per stress type.

In total, 42 articles address this topic, 31 based on culture-dependent techniques, 10 based on culture-independent techniques and 1 considers both (Figure 3a). The most studied environmental stresses are drought (15 articles in total), nutrient availability (9 articles), pollution (7 articles), and soil salinity (6 articles) (Figure 3b). While most articles center on single stress (31), articles considering multi-stresses are rare (Table S3). Culture-dependent studies focus on drought, nutrient availability, and soil salinity. Their experimental approach involves exploring the PGPM capacity to mitigate the stress effect in the plant host. Particularly, drought and soil salinity are considered the major stresses for agriculture, which lead to significant yield reduction [31,32]. Therefore, biotechnological solutions, as projected by the authors of these studies, would be relevant for the productive sector but often fail to advance from the laboratory scale to actual commercialization by the industry [33].

For the culture-independent studies, the range of investigated environmental stresses is much broader (Figure 3b). In most cases, the research aims to understand microbial diversity, adaption and/or variability in a natural environment without a specific interest in its applicability. Despite not being directly applicable, this knowledge is valuable for understanding microbial sensibility to different conditions, which, e.g., can help to develop or improve the management of agricultural soils. 

### 3.3. Towards a Biotechnological Solution

**Figure 4:**
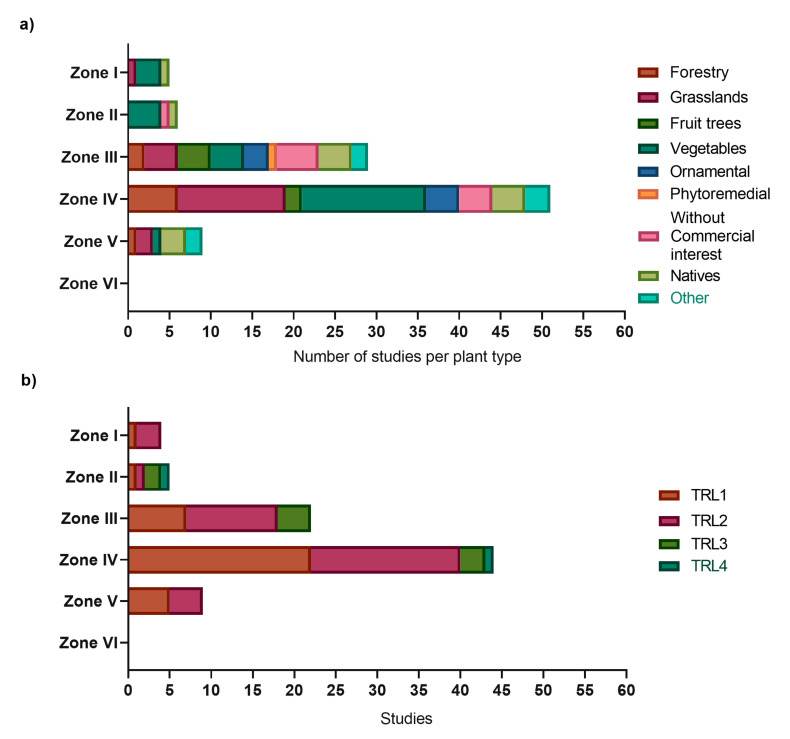
Biotechnological perspective of PGPM studies (only considering culture-dependent studies with microbial inoculation). (**a**) plant types used in PGPM studies (studies can account for more than one plant type), and (**b**) technology readiness levels obtained in the study. (For the description of zone I to zone VI, please refer to methodological Section 2.4).

In this section, only culture-dependent studies working with microbial inoculation (single strain and/or consortia) were considered, which sum 84 articles. Of these, 55 studies used single-strain inoculation; in 29 studies, microbial consortia were applied. Most biotechnological studies were conducted in the warm-temperate and Mediterranean zone (zone III and IV) (Figure 4). In these two zones, researchers can take advantage of the high ecosystem and crop diversity reflected in a higher diversity of investigated plant types (Figure 4a). However, increasing drought due to climate change will affect this area [34] and research from arid zones (zone I and II), anticipating climate change consequences in formerly more humid areas is urgently needed, but much less performed. The studies from arid zones (zone I and II) mostly focus on vegetable crops, such as tomato and lettuce, which are commonly cultivated despite their water demand. In zone V, PGPM inoculation in grasslands and forest trees is investigated, reflecting the dominant land use types. Finally, no studies with microbial inoculations were found for the subantarctic zone (zone VI).

The relatively high number of studies conducted with vegetables and grasslands reflects these crop plants’ commercial importance (local and global) [35]. Nonetheless, the prevalence of these crops vs. fruit trees or forest trees in scientific studies might also be influenced by their growth characteristics, e.g., short life cycle, plant size, and availability of physiological and genetic information for the species. Such arguments gain importance when considering framework conditions in science, which are defined by the duration of research projects, availability of funds, and scientific excellence measured in publications [33].

During the biotechnological development process, the proof of concept for a new technology is an important step (TRL 3). At that level, the scientific knowledge is considered sufficiently founded to encounter the validation and scaling to the operational environment [36]. In this section, 87% of the articles were classified as TRL1 or 2 (36 and 37, respectively), reflecting knowledge generation in the context of PGPM and their interaction with the plant host (Figure 4b and Figure S3). Only 11% of the articles corresponding to TRL3, and the remaining 2% report findings equivalent to TRL4. These findings exemplify a diagnosis published by other authors in the field [36,37,38]. Among others, the lack of funding for more applied research is probably the major limitation, despite the effort made by the Chilean state to promote this transitional stage via special calls of the national science agency and tax breaks for companies involved in research and development (R+D). It is important to mention that research beyond TRL3 rarely is published in scientific journals, in order to conserve data privacy for patenting (or other form of protection of the intellectual property) [33]. Additionally, the formulation of PGPM based products and the scaling up requires collaborative research, e.g., with bioengineers, agronomists, commercial engineers etc., and alliances with the productive sector, which often escape the interest (or possibilities) of a scientist in PGPM [36,37].

**Figure 5:**
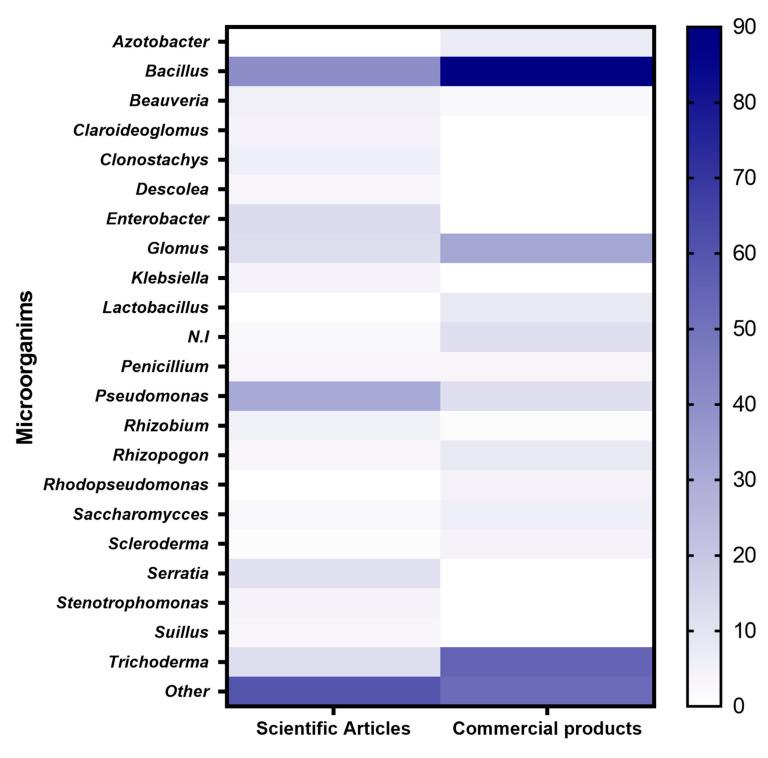
Microorganisms described in scientific publications and used in commercial products in Chile (Data Source: SAG, Chile; Table S4).

Another relevant aspect is the selection of the microbe to be investigated o scaled up. Here, arguments for science and industry differ widely from each other. For science, the novelty of the research and cultivability of the microorganism is often decisive, whereas for industry technical, economic, and even social aspects (sustainability, innocuousness) are relevant [33]. The higher degree of freedom of choice is reflected in a higher taxonomic diversity of the study microorganisms in science (Figure 5). Interestingly, *Bacillus* sp. strains are the most used microorganisms in science and industry. Many commercial products contain *Bacillus* sp. strains, as single strains or consortia (often with *Trichoderma*) (Supplementary Material 2), because they maintain high levels of viability in formulations and relatively long shelf life [7,36,39] such as *Serratia* spp. and *Klebsiella* spp. are only found in scientific articles, and despite their promising plant growth promotion effects, such strains are difficult to transfer to the industry area in generally stigmatized as pathogens [30,36].

## 4. Gaps in Knowledge and Technology Transfer of PGPM Research

The knowledge and technology transfer around biotechnological solutions based on PGPM between academia and industry is limited [36,40]. During our analysis of the situation in Chile as a study case, we discussed some critical points of the transfer process, e.g., scaling up from the proof of concept to a proven technology includes various pitfalls [37,41], deviating selection criteria for the PGPM study objects [33]. In this section, we aim to put our results into a broader context, focusing on three transversal aspects: inter- and transdisciplinary research, territories, public policy and society, and collaborative innovation between academia and industry.

### 4.1. Inter- and Transdisciplinary Research

We detected that PGPM research mainly focuses on understanding interaction mechanisms, microbial community assembly, and function. However, this research rarely leads to application. We consider that a low level of collaboration with other disciplines might be involved, as other scientific specialties are needed during the transfer process. In this sense, inter- or transdisciplinary science provides a possibility to overcome subject-specific limitations. At the same time, scientific findings are communicated in a way that is easier to understand and accessible to various stakeholders (such as industrial partners) [13]. In the health sector, incorporating interdisciplinary formation improved the innovation in research and increased the interaction between professionals of different areas to resolve problems with input from different disciplines [42]. The major challenges for inter- or transdisciplinary science are developing a common base for communication and understanding, generating a common conceptual model, and appropriately handling expectations and misunderstandings.

Beyond the purely academic context, transdisciplinary collaboration is an opportunity to integrate the local and regional community in participatory research, allowing it to properly address real-world problems and sustainable development goals [43]. Additional benefits of such a participatory approach are promoting an open-minding social attitude, enabling cross-learning between participants, and building networks [13,33].

To achieve this, the availability and distribution of funding resources become relevant. They must be adapted to current requirements [44] to ensure an adequate environment for cutting-edge innovations and their application and egalitarian access to knowledge and development in societies.

Inter- and transdisciplinary collaborations in science allow greater knowledge integration and comprehension of the proposed study subject [45]. This also applies to underexplored geographic areas in our review (e.g., arid and semi-arid zones), where PGPM science is much less conducted but highly relevant to face climate change and the increase in desertification worldwide. Similarly to Vásquez-Dean et al. (2020) [46], we consider that this global challenge requires and demands a stronger linkage and cooperation between researchers worldwide.

### 4.2. Territories, Public Policy and Society

The geographic distribution of the PGPM studies in Chile reflects a tendency that has been observed as a general pattern with regard to research efforts on soil biodiversity (and microbial diversity studies): most studies are concentrated in temperate systems, from which microbial diversity and ecological function often are extrapolated to other ecosystems [47,48]. Moreover, as mentioned before, much less research has been published from arid and semiarid ecosystems which is worrying particularly in the context of increasing desertification due to global change [46,49].

**Economic and infrastructure development** of a country strongly correlates with establishing national scientific capabilities [50]. For instance, the economic development in most dryland countries is very low compared to countries in other ecosystems [51,52], which could explain (at least partially) the lower scientific productivity in these regions. Emerging and developing countries generally identified the potential of innovation and technology transfer to advance economic development and employment. In this context, allocating public funds is the main source for establishing local capability and sustainable science systems [53,54]. Take the example of Chile (despite being an OECD member state), where an average of 0.36% of GDP (Gross Domestic Product) is inverted in science, and only 33% of this amount is contributed by the private sector, which is far below OECD average [55]. Under such conditions, scientific research in emerging and developing countries intends to compete with developed countries, which is not yet achieved but is on track [50]. In addition, the research hotspots of a country geographically correlate with its economically most competitive regions, generating a high degree of inequality in the national science system in both developed and developing countries [50].

**Public science policy** in developing countries seeks to strengthen knowledge and technology transfer via different mechanisms, focusing on generating public incubators to support entrepreneurship and startups [33,53]. This strategy is also related to achieving the UN Sustainable Development Goals, and both are strongly based on science, technology, and innovation. Public incubator programs successfully contributed to economic and social development in countries such as India, Brazil, or China, whereas African countries just started with such public programs [53]. A challenge for policymakers is how to invert public funds most effectively to locally or regionally in order to achieve a sustainable development [43]. Therefore, an overall strategic vision for the territory based on a bottom-up and inclusive process involving all stakeholders and a comprehensive diagnosis of territorial strengths and challenges is required [56].

**Public awareness**. Despite the multiple benefits PGPM based biotechnological solutions offer to the farmer as well as to the final consumer, these are little integrated into crop management, and commonly little is known about them. At least since the pandemic, microorganisms have become part of daily awareness, but mainly as pathogens to humans, animals, or plants. This negative image, also supported by public health and the advertisement of cleaning and hygienic products that eliminate up to 99.9% of microbes, causes rejection and misunderstanding of microorganisms and their importance for us as well as all ecosystems on earth [57,58]. Therefore, it is important to convert their bad reputation through education (schools and universities) and outreach programs, highlighting the benefits generated by microorganisms (e.g., pharmacy, health, food production, soil fertility, etc.) [57,59]. In the context of PGPM for agriculture, communication and training with specific information and practical guides for using microbe-based products could contribute to overcoming uncertainties, such as efficiency, specificity, or compatibility with other products (e.g., fertilizers, pesticides) [10].

### 4.3. Collaborative Innovation between Academia and Industry

Although the number of articles analyzed in this review is not small (n > 200), their impact on society is not significant. Kampers et al. (2022) identified in their study two mayor causes for the lack of market introduction: ineffective communication (often leading to the loss of opportunity) and the differences of TRL that academia and industry operate on (which is linked to the aims and expectations on both sides). This divergence of objectives in science and the productive sector could also be complex for the agricultural industry [60].

In the context of ineffective communication, other studies confirm that the dissemination of scientific knowledge historically occurred through platforms, which are not suitable for technology transfer [61]. An “economic impact can be achieved only after a complex, temporally unfolding sequence of interactions between formal and informal channels of knowledge transfer” [61].

We consider that the generation of a collaborative innovation environment, where academia as well as public and private stakeholders (including the local community) take part, is crucial to enhance the acceptance and integration of PGPM on the way to sustainable agriculture. The establishment of such collaborative network is still in its infancy in many countries and is not necessarily linked to the economic potential of a country, as the example of Chile shows [55,62]. In their revision of co-invention patents in Chile, Pinto et al. (2019) reveal that the inventor network in Chile is highly fragmented and a significant majority of the patents granted in Chile belong to foreign inventors. 

Such studies highlight the need to strengthen the science-policy–society interface and the collaborative generation of knowledge, based on renewed public policy frameworks, focusing on the local and national scale first [63,64].

In a future scenario, dynamic system would arise in which a rather industrial sector of society generates demand for innocuous agricultural products, stimulating the growth of the biotechnological market, such as the manufacture of biostimulants and biofertilizers based on PGPM. Thus, dynamic and growing marketing with costs and benefits arises. Society generates a demand as the final consumer of safe agricultural products, which generates a growing market for PGPR products, leading to the establishment of costs/benefits for the producer.

## 5. Final Remarks

Since the discovery of the benefits that the interaction of plants and beneficial microorganisms can generate for agriculture sustainability and adaption to climate change challenges, much scientific research has been developed, and knowledge of involved mechanisms has been gained. However, this scientific knowledge about PGPM does not penetrate society, and technology transfer to the productive sector (agriculture, forestry, and biotechnological industry) is low. This transfer from the academia to the productive sector is essential to scale up PGPM based solutions for agriculture. The bridge of effective communication has two heads and crossing requires the commitment of both. Neither academia nor industry can meet unfounded expectations during technology transfer, but mutually clarifying their needs, capabilities, and limitations is the starting point for successful collaborations. In this context, intermediaries such as R & D bureaus, or even startups, together with a strategic public policy, could contribute to overcoming industry’s limited experience in collaborative innovation.

A more efficient funding mechanism is needed to bridge the gap between scientific findings and promising prototypes, promoting a collaborative environment between academia and industry. Regionally developed PGPM solutions for specific soil and climate conditions offer the opportunity to diversify the productive regional sector, improving the labor market and social development.

Awareness raising in society and within the agricultural sector should be reinforced. Involving society with arguments on food quality and safety and sustainable crop management could be a powerful tool to enhance the incorporation of PGPM based solutions in agriculture. In addition, the current public interest in the circular economy enables the possibility of bringing PGPM to a wider audience and winning new partners.

## Data Availability

The data is available in the Supplementary Material 2.

## Supplementary Materials

The following supporting information can be downloaded at: https://www.mdpi.com/article/10.3390/microorganisms11041061/s1. The following supplementary information can be downloaded at: Supplementary Material 1, containing Figure S1: scheme of the methodology of obtaining the metadata; Figure S2: number of scientific publications in the time according to the technique of studies that used: Dependent culture (orange), independent culture (sky blue), and both techniques (green); Figure S3: Distributions of studies according to agroclimatic zone by Knowledge generation and application; Table S1: Descriptions of the agroclimatic zone of Chile; Table S2: number of articles by agroclimatic zones and sty approach; Table S3: Number of articles according to the number of mechanisms PGPM described; Table S4: Microorganisms described in scientific publications and used in commercial products in Chile (Suerce: SAG, Chile). Supplementary Material 2: Meta-data file with the revised articles and the extracted information used in this study.
