# Supplementary material for: Challenges for Plant Growth Promoting Microorganism Transfer from Science to Industry: A Case Study from Chile
Source: Microorganisms. 2023 Apr 19;11(4):1061. doi: 10.3390/microorganisms11041061 (PMC10140820; doi:10.3390/microorganisms11041061)
Supplement: Supplementary file 1 [file microorganisms-11-01061-s001.zip › SM1.pdf]

Suplementary material 1

Knowledge transfer gaps from science to the productive sector: Beneficial plant-microorganism interaction

Eduardo Muñoz-Carvajal[1,2], Juan Pablo Araya-Angel[1,2], Nicolás Garrido-Sáez[1,3], Máximo González[1,4], Alexandra Stoll[1,4]*.

1. Methodology

Obtentions of de metadata

A bibliographic search of articles published up to May 2021 was performed in the main databases for the stipulated purposes, such as Pubmed, WOS, and Scopus, without language restriction. In addition, articles manually added that did not arise from the algorithm search in the indicated databases were added manually. The manual selection of articles was based on the trajectory of known research groups that should be present for the subsequent meta-analysis (Figure S1).

For database searches, the following keyword combinations were used:

1. (bacteria* OR fung* OR microorganism* OR mycorrhiza*) AND (Chile OR chilean) AND plant)

2. (Bacteria* OR (Fung* OR (microorganism* OR (Mycorrhiza* OR ((Microbi* OR (rhizo*) AND ((Plant*) OR AND ((Chile) OR (Chilean) OR (Atacama) OR (Andes) OR (Andean) OR (Chilean environment*) OR (Chilean highland*).

Reviews were excluded from the database, incorporating articles that meet the following criteria: (1) the microorganisms must be described, isolated and/or applied in Chile, (2) a beneficial microorganism-plant interaction must be described in the article (excluding plant pathogens) (Figure S1).

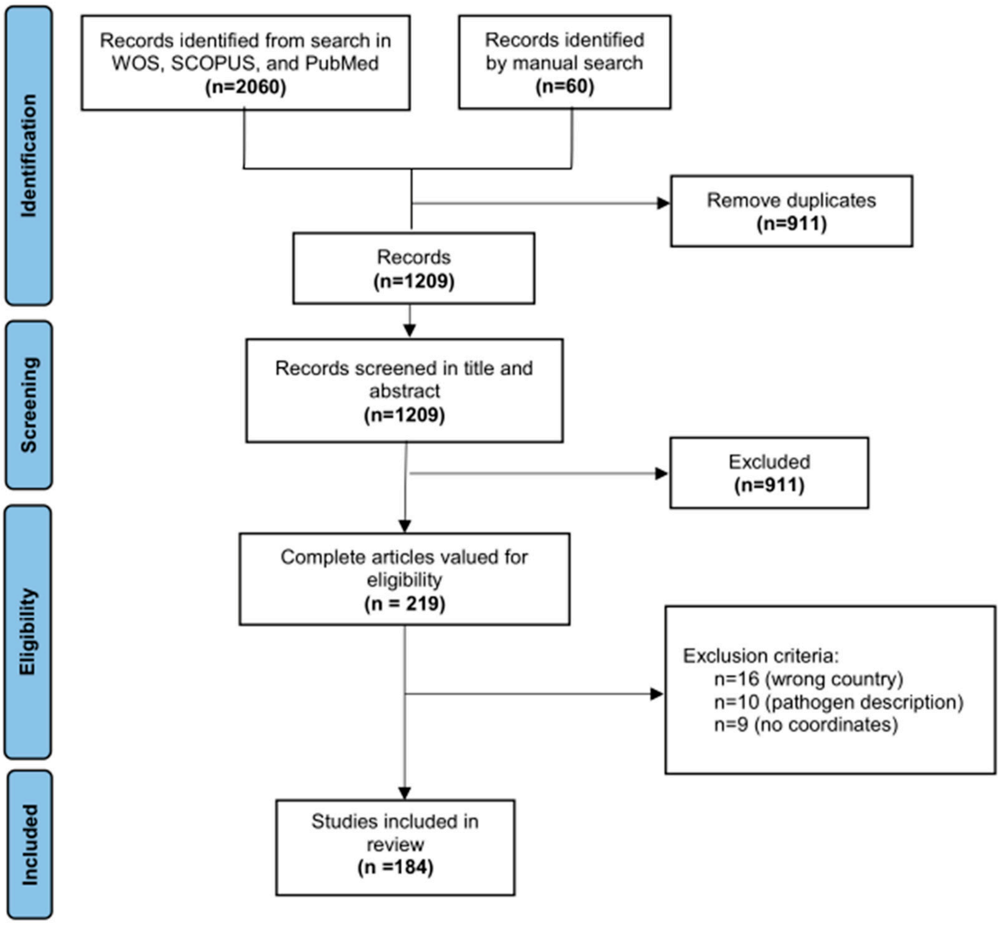

Figure S1: scheme of the methodology of obtaining the metadata.

Table S1: Descriptions of the agroclimatic zone of Chile.

Agroclimatic Zones	Descriptions
Zone I	It comprehends the region de Arica y Parinacota, Tarapaca, and Antofagasta. In this zone predominant, water shortage because of low precipitations. Your landscape is arid, with a low presence of vegetation.
Zone II	It comprehends the region of Atacama and Coquimbo. This zona transitions zones between zone I (arid Conditions) and zone III with Mediterranean conditions. For this, the present condition between arid and semiarid. The geographic relief and orography of the zone permit the development of agriculture.
Zone III	It comprehends the Region of Valparaiso, Metropolitana, Del Libertador Bernardo O'Higgins, and Maule. These zones present Mediterranean climatic conditions. Due to the increased humidity of the zone, the vegetation increases compared to Zone II. Because of the climatic and soil conditions, this zoned present important agriculture with no native crops.
Zone IV	It comprehends the Region of Ñuble, BioBio, and La Araucania. This zone presents a climatic degradation, with the north first for the dry Mediterranean to finish the humid Mediterranean conditions in the south. It is characterized by irrigations proportionated by rivers Itata, Biobio, Imperial, and Tolten
Zone V	It comprehends the Region of Los Ríos y Los Lagos. Present a rainy influence on the vegetation type rainforest and Valdivian rainforest. The presents of rivers and lakes with gentle slopes characterize the hydrography of the zone.
Zone VI	It comprehends the Region of Aysen del General Carlos Ibañez del Campo and de Magallanes y Antártida Chilena. Its clime is characterized by low temperature and fort wind.

References

Santibáñez, F., Santibáñez, P., Caroca, C., & González, P. (2017). Atlas Agroclimático de Chile. Universidad de Chile. Santiago, Chile.

Results

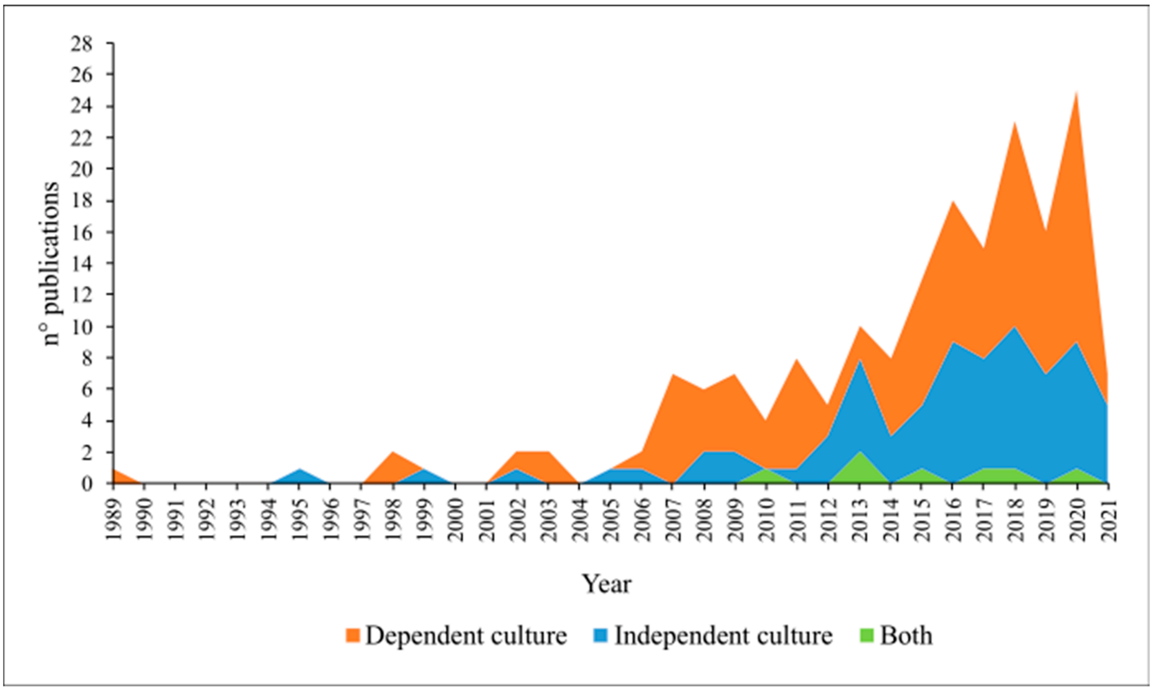

Figure S2: number of scientific publications in the time according to the technique of studies that used: Dependent culture (orange), independent culture (sky blue), and both techniques (green).

Table S2: number of articles by agroclimatic zones and study approach

Agroclimatic Zones	Dependent Culture	Independent Culture	Both	Total
**I**	9	9	0	18
**II**	7	5	0	12
**III**	23	14	1	38
**IV**	54	24	5	83
**V**	9	11	1	21
**VI**	3	8	0	11
**total**	105	71	7	183

Table S3: Number of articles according to the number of mechanisms PGPM described.

Number of mechanism PGPM	Number of articles	Percentaje of articles
**1**	154	84,1
**2**	15	8,2
**3**	7	3,8
**4**	3	1,6
**5**	2	1,1
**6**	2	1,1

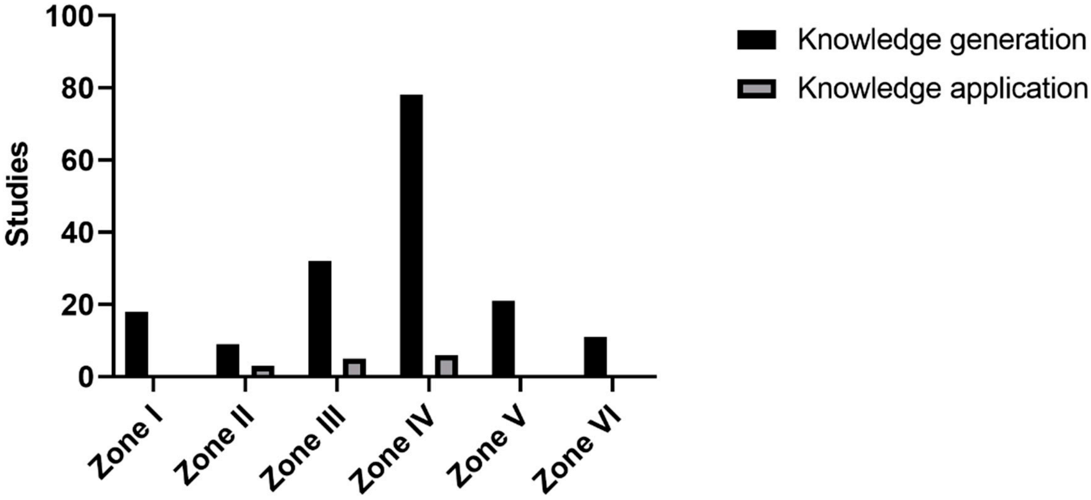

Figure S3: Distributions of studies according to agroclimatic zone by Knowledge generation and application.

Table S4 : Microorganisms described in scientific publications and used in commercial products in Chile (Suerce: SAG, Chile).

	Scientific Articles	Commercial products
**Azotobacter**	0	7
**Bacillus**	40	90
**Beauveria**	5	2
**Claroideoglomus**	4	0
**Clonostachys**	6	0
**Descolea**	3	0
**Enterobacter**	13	0
**Glomus**	12	32
**Klebsiella**	4	0
**Lactobacillus**	0	8
**N.I**	2	12
**Penicillium**	3	3
**Pseudomonas**	31	12
**Rhizobium**	5	1
**Rhizopogon**	3	8
**Rhodopseudomonas**	0	4
**Saccharomycces**	2	6
**Scleroderma**	1	4
**Serratia**	11	0
**Stenotrophomonas**	4	0
**Suillus**	3	0
**Trichoderma**	12	55
**Other**	60	52
